# Dual snare endoscopic extraction of a gastric foreign body using a dual‐channel endoscope

**DOI:** 10.1002/deo2.70065

**Published:** 2025-01-23

**Authors:** Amitjeet Singh, Edward Young, Rajvinder Singh

**Affiliations:** ^1^ Department of Gastroenterology Lyell McEwin Hospital Elizabeth Vale Australia

**Keywords:** dual‐channel endoscope, dual snare method, endoscopic retrieval, foreign body ingestion, push‐pull technique

## Abstract

Foreign body ingestion, though rare, poses significant health risks, with 10%–20% of cases requiring endoscopic intervention. This article presents a novel case of a 16‐year‐old female who ingested a cigarette lighter, leading to abdominal pain and radiographic confirmation of a gastric foreign body. Initial attempts at retrieval using grasping forceps and snares were unsuccessful due to the object's size and shape, risking potential complications. We employed a dual‐channel endoscope utilizing a push‐pull technique with two snares, one capturing the blunt end and the other the sharp edge of the lighter. This method facilitated safe extraction through the gastroesophageal junction without causing trauma. This case highlights the efficacy of dual snares in the endoscopic retrieval of challenging foreign bodies and emphasizes the importance of appropriate techniques in preventing complications during such procedures.

## INTRODUCTION

Foreign body ingestions are rare but can pose serious risks. Most pass naturally, but 10–20% require endoscopic removal, and 1% need surgery.^1^ Ingested foreign bodies can include organic material, such as food boluses, or non‐organic objects, such as coins, toys, magnets, or batteries. Foreign bodies are commonly removed endoscopically using forceps, snares, baskets, and nets.^2^ The physical characteristics of ingested objects greatly influence the available techniques for safe and atraumatic endoscopic retrieval. Long and pointed foreign objects in the stomach are challenging to remove endoscopically and can cause complications such as tears or even perforation.^2^ The removal of long and sharp foreign objects has previously been described using a dual‐snare technique using two endoscopes either simultaneously or successively.^3^, ^4^ We present a novel case highlighting the use of dual snares with a dual‐channel endoscope to safely maneuver a gastric foreign body using a push‐pull technique.

## CASE PRESENTATION

A 16‐year‐old female with a history of anxiety and depression had purposefully ingested a cigarette lighter 3 weeks prior to presentation. She presented to the hospital due to ongoing abdominal pain over two days duration. There were no significant findings on clinical examination, and laboratory investigations were unremarkable. An X‐ray of the patient's abdomen showed a radiopaque foreign body in the left upper quadrant of the abdomen, likely within the gastric body.

**FIGURE 1:**
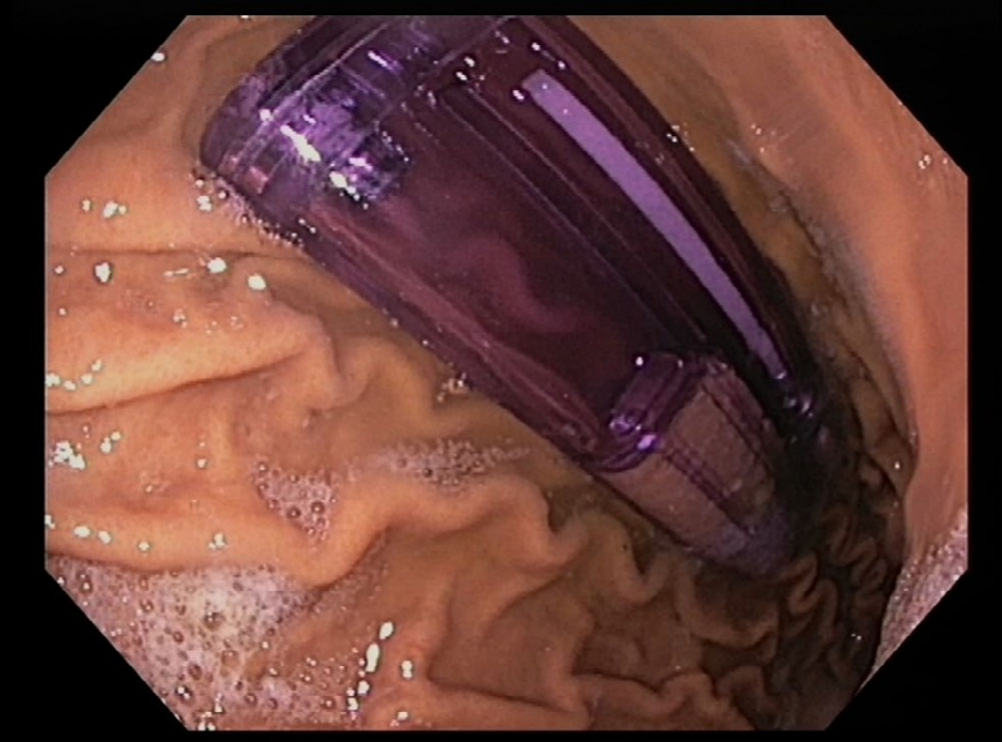
Endoscopic view of lighter within the stomach.

At endoscopy, there was a single cigarette lighter in the gastric body with the metal cap removed (Figure 1), exposing a sharp plastic edge. Initially, grasping forceps were used but these would not capture the lighter adequately to traverse the lower esophageal sphincter. A single snare was used but this positioned the lighter perpendicular to the esophageal lumen when the snare was tightened which would have made retrieval dangerous. An attempt with an overtube was also unsuccessful as the lighter was too large. The use of a Roth Net was not attempted as it would not allow for appropriate orientation and could have led to difficulties in releasing the lighter if it got stuck.

**FIGURE 2:**
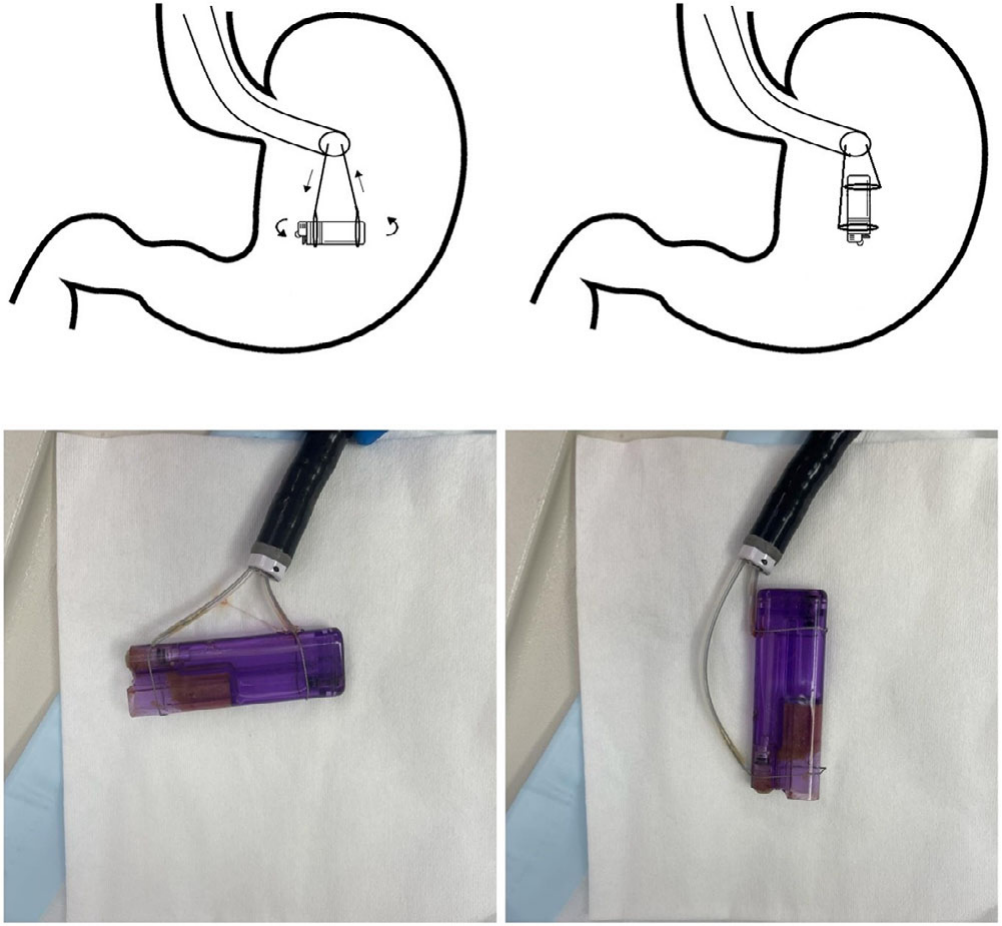
Changing the position of the lighter within the stomach utilizing a push‐pull technique using a dual‐channel endoscope.

A dual‐channel gastroscope was then used, which facilitated the careful positioning of the lighter using two snares. One snare was placed over the rounded edge of the lighter, which was positioned cranially, while the second snare captured the sharp edge of the lighter and was positioned caudally using a push‐pull technique (Figure 2). The lighter could then be gradually pulled through the gastroesophageal junction and out of the mouth without complication. The scope was re‐advanced after removing the lighter and there was no significant trauma. The total duration of the procedure was 60 min, consisting of 10 min for anesthesia and intubation, 45 min for the trial of other techniques and the time taken to perform the two‐snare technique was 5 min.

## DISCUSSION

This case demonstrates the use of dual snares through a dual‐channel endoscope to successfully remove a long and sharp object from the stomach using a push‐pull technique. Conventional methods of removing such objects involve using a single device (snare, basket, forceps, and net) with a protective element (overtube and hood).^5^, ^6^ Procedural challenges to removing such an object involve positioning the to prevent perforation or impaction during extraction, orienting the object's long axis in the extraction line, and applying traction to the foreign body.^3^ Strategies for success using our technique would rely on securing both ends using the snares, with one snare placed over the blunt end of the lighter, positioned cranially, and the second snare placed over the sharp end, positioned caudally. This would be followed by a coordinated push‐pull movement by pushing the caudal snare downwards and the cranial snare upwards. Candidates for our described technique would be sharp objects (broken pieces of hard plastic, needles, and nails), rod‐shaped objects (pens, toothbrushes, and dining utensils), and asymmetrically shaped objects. Less suitable candidates would include circular/ spherical objects (coins and balls), and soft compressible objects (Video S1).

The use of a push‐pull technique using two devices has previously been described with other endoscopic accessories (balloons and snares); however, to our knowledge, it has not previously been described using dual snares with a dual‐channel endoscope.^3^, ^4^, ^7^ The advantage of our described technique using two snares is with regards to its applicability to objects that are long, sharp, or asymmetrical. In addition, it allows for enhanced control of the movement of such objects preventing them from turning perpendicular to the esophagus and thus minimizing the risk of impaction or perforation and reducing the overall procedural time. It would however require the use of a dual‐channel endoscope which may not be readily available at all centres.

## CONFLICT OF INTEREST STATEMENT

None.

## ETHICS STATEMENT

Informed consent was obtained from the patient and her legal guardian prior to the publication of this report. All patient identifiers have been removed to ensure confidentiality.

## Supporting information



Video S1 Demonstration of technique.
